# On a New Styptic

**Published:** 1892-01-30

**Authors:** 


### SURGERY.

### ON A NEW STYPTIC.

The tendency of recent times has been to discredit the value of the class of remedies called styptics. In modern surgery torsion, ligatures, and carefully applied pressure have largely superseded them, and for internal hæmorrhage more reliance is placed on ice and ergotin, than on the time-honoured compounds. Even in epistaxis inflation instruments, or a soft rubber catheter, over which an elastic bag is tied, can be introduced in a moment, inflated, and secured so as to form a painless substitute for styptics, or the troublesome plug in the posterior nares. Still we are greatly in need of more efficient means of staying sudden hæmorrhage when cold, pressure, elevation of the part, or thorough surgical treatment is applicable. Hammamelis, in its various forms, would appear to be valuable chiefly in venous hæmorrhage, but for arterial hæmorrhage from the lungs, intestinal tract or uterus, as well as for use in less serious bleeding as a first aid treatment we still need a reliable hæmostatic. In connection with this some investigations recently published by Dr. A. S. Wright, are of great interest. He has examined the effect on coagulation of the addition of fibrin ferment and calcium salts, and in doing so shows once more the practical value of scientific physiology. He obtains the ferment by whipping blood diluted with three volumes of water, washing the fibrin clots for ten minutes, and then leaving them in five volumes of clean water for twenty-four hours. The clots may be sterilised by keeping in strong alcohol, or a solution of carbolic acid, may be added to the watery extract if required. The extract in any case has added to it one per cent. of calcium chloride. Lime salts have not been usually employed as styptics, but rather as astringents in diarrhœa and mucous discharges. However, it has been proved that they exert an important influence over the coagulation of the blood. By employing externally the mixture of calcium chloride and the extract of fibrin ferment, Wright was able to control the bleeding from even large vessels in animals in a most remarkable manner, and to reduce the time required for coagulation of blood when poured into test tubes by about one-half. Equally satisfactory results were given in an operation on the cervix uteri in the human subject, where there was a difficulty in controlling the bleeding by ligatures.

There appears to be no possibility of stopping hæmorrhage by the injection of fibrin ferment or tissue fibrinogen, but the administration of large doses of calcium salts by injection or by the mouth gave some surprising results. When all the small vessels on one side of a dog's neck were cut across, the blood clotted as it escaped and formed a tough layer, which stopped the hæmorrhage completely. The calcium chloride, when administered by the mouth required about 30 to 45 minutes to produce its full effect, as shown by the time taken to coagulate samples of the blood drawn off at intervals. On the other hand, no intravascular clots were discovered, and some effect on coagulation was attained in even a few minutes. Dr. Wright remarks that the doses he employed with animals were very large, equal to four drachms of calcium chloride in a pint of water if administered to a human being, but probably a smaller amount would effect the end in view. It is doubtful how far small repeated doses in cases of threatened hæmorrhage would act, for the salt is rapidly excreted by the kidneys, but the whole subject is of such importance, and it demands thorough investigation. If his results prove to be applicable to the human subject, the researches of physiologists will have given us a powerful remedy in some of the most terrible and hopeless situations which our patients can meet with. In such investigations, however, there are numerous sources of error, but it seems probable that his cautious and well-considered experiments may eventually provide a valuable hæmostatic and possibly even a remedy for certain forms of dropsy.

